# Sickle Cell Disease and Dental Care Access Among Medicaid-Enrolled Youths

**DOI:** 10.1001/jamanetworkopen.2025.29849

**Published:** 2025-09-03

**Authors:** Ashley Kranz, Hannah K. Peng, Allison A. King, Sarah J. Clark, Melissa A. Plegue, Sarah L. Reeves

**Affiliations:** 1 RAND Corporation, Arlington, Virginia; 2 Susan B Meister Child Health Evaluation and Research (CHEAR) Center, Department of Pediatrics, University of Michigan, Ann Arbor; 3 Division of Pediatric Hematology/Oncology, Department of Pediatrics, Washington University School of Medicine, St Louis, Missouri; 4 Department of Epidemiology, University of Michigan, Ann Arbor

## Abstract

This cross-sectional study compares receipt of dental care among youths with and without sickle cell disease in Michigan.

## Introduction

Sickle cell disease (SCD), an inherited blood disorder associated with significant morbidity and early mortality, is diagnosed in approximately 2000 newborns annually. There exists a reciprocal relationship between oral health and SCD. Dental caries may lead to an infection, which may trigger a sickle cell crisis.^1^

SCD increases risk of dental complications, which emphasizes the importance of early preventive services among youths.^2,3^ This study examined receipt of dental services for youths with SCD benchmarked against the general population of Medicaid-enrolled youths in Michigan.

## Methods

The University of Michigan institutional review board determined this cross-sectional study to be not regulated, as it is public health surveillance. We followed the STROBE reporting guideline. Among youths aged 1 to 20 years, SCD was identified using validated case definitions through the Michigan Sickle Cell Data Collection (MiSCDC) program.^4^ Claims were identified for any dental services, preventive dental services, and dental treatment (eMethods in Supplement 1). Counts for these services were obtained from the Centers for Medicare & Medicaid Services data for all youths enrolled in Medicaid in Michigan in 2022.^5^ Analyses were conducted from January to June 2025.

Proportions of youths receiving each service type were calculated overall and by age group, and compared between data sources using χ^2^ tests. *P* < .05 was considered statistically significant, and all tests were 2-tailed. R version 4.5.1 (R Project for Statistical Computing) was used for all analyses.

## Results

The sample consisted of 1096 youths aged 1 to 20 years with both SCD and Michigan Medicaid, and 1 181 391 youths aged 1 to 20 years with Michigan Medicaid. More than 40% of youths in both groups were aged 6 to 14 years (482 children with SCD [44.0%]; 541 531 in the pediatric Medicaid population [45.8%]). Compared with youths with SCD, the pediatric Medicaid population had similar rates of any dental services (42% vs 44%), preventive dental services (38% vs 40%), and dental treatment (15% vs 18%) (Figure). When examined by age group (Table), most dental service rates were higher, although not significantly different, in the Medicaid population. For example, among youths aged 6 to 14 years, those with SCD were significantly less likely than the Medicaid population to receive any dental services (50% vs 54%; *P* = .049) and preventive dental services (46% vs 51%; *P* = .047). However, children aged 1 to 5 years with SCD had higher rates of receipt of any dental services (38% vs 34%; *P* = .17) and preventive dental services (36% vs 31%; *P* = .06) compared with the pediatric Medicaid population, although the differences were not statistically significant.

**Figure.**
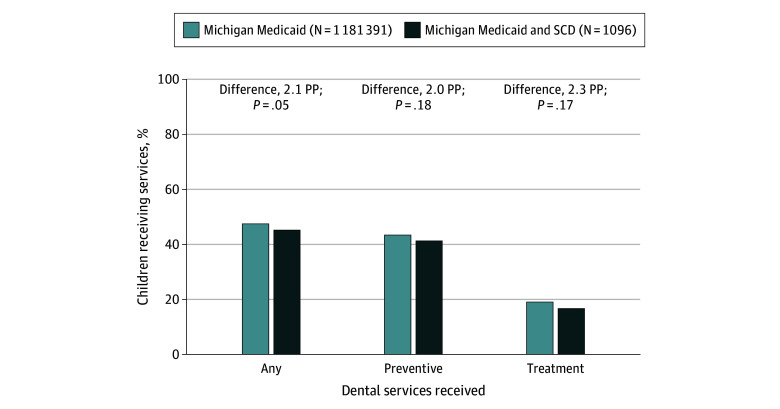
Receipt of Dental Services for Youths With Medicaid and Sickle Cell Disease (SCD) in Michigan in 2022 PP indicates percentage points.

**Table.** Receipt of Dental Care for Children With Medicaid and Children With SCD and Medicaid in Michigan, 2022^a^

Receipt of dental care by age group	No. (%)		Difference, percentage points	*P* value^b^
	Michigan Medicaid and SCD (n = 1096)	Michigan Medicaid (n = 1 181 391)		
**Age 1-5 y**				
No.	317	309 877	NA	NA
Any dental services	119 (37.5)	104 456 (33.7)	3.8	.17
Preventive dental services	114 (36.0)	95 974 (31.0)	5.0	.06
Dental treatment services	19 (6.0)	24 368 (7.9)	−1.9	.26
**Age 6-14 y**				
No.	482	541 531	NA	NA
Any dental services	239 (49.6)	293 310 (54.2)	−4.6	.049
Preventive dental services	223 (46.3)	275 568 (50.9)	−4.6	.047
Dental treatment services	97 (20.1)	124 752 (23.0)	−2.9	.14
**Age 15-20 y**				
No.	297	29 983	NA	NA
Any dental services	103 (34.7)	123 915 (37.6)	−2.9	.34
Preventive dental services	84 (28.3)	106 147 (32.2)	−3.9	.17
Dental treatment services	53 (17.8)	59 887 (18.1)	−0.3	.95

Abbreviations: NA, not applicable; SCD, sickle cell disease.

^a^ Data for youths with Michigan Medicaid and SCD are from claims. Data for Michigan Medicaid is from the Centers for Medicare & Medicaid Early and Periodic Screening, Diagnostic and Treatment Annual Reporting Data Files.

^b^ *P* values are from χ^2^ tests comparing proportion of individuals receiving each type of service between data sources.

## Discussion

In this cross-sectional study of youths with Medicaid in Michigan, fewer than half of youths with SCD (42%) received dental services in 2022. All Medicaid programs cover pediatric dental care, and all youths are recommended to receive annual dental visits, yet youths with Medicaid had low rates of dental care, regardless of SCD status. Youths with SCD in Michigan had comparable rates of dental service usage to the general pediatric Medicaid population, which is concerning given their risk for dental complications.^1,2,3^ These findings underscore the need for targeted interventions, such as enhanced training to ensure dentists are willing to care for youths with SCD and for medical clinicians to understand the importance of dental care for youths with SCD. However, low participation of dentists in Medicaid coupled with the need for more guidance and protocols to inform the delivery of dental care to youths with SCD may hinder these efforts.^3,6^ As the drivers of low rates of dental care are multifactorial, future research should focus on identifying and addressing specific barriers to optimal dental care in this vulnerable population.

One limitation to this study is that the rates of dental service receipt for youths with Michigan Medicaid include youths with SCD. However, because fewer than 0.1% of youths in Michigan Medicaid are in MiSCDC, this inclusion is unlikely to impact the overall rates. Given increased risk of oral health problems among patients with SCD ,^1,2^ strategies are needed to expand dental services for youths with SCD.

## Notes

Supplement 1.
eMethods.

Supplement 2.
Data Sharing Statement
