# Supplementary material for: Sickle Cell Disease and Dental Care Access Among Medicaid-Enrolled Youths
Source: JAMA Netw Open. 2025 Sep 3;8(9):e2529849. doi: 10.1001/jamanetworkopen.2025.29849 (PMC12409594; doi:10.1001/jamanetworkopen.2025.29849)
Supplement: Supplement 1. — eMethods. [file jamanetwopen-e2529849-s001.pdf]

Supplemental Online Content

Kranz A, Peng HK, King AA, Clark SJ, Plegue MA, Reeves SL. Sickle cell disease and dental care access among Medicaid-enrolled youths. *JAMA Netw Open.* 2025;8(9):e2529849. doi:10.1001/jamanetworkopen.2025.29849

eMethods.

This supplemental material has been provided by the authors to give readers additional information about their work.

© 2025 Kranz A et al. *JAMA Network Open*.

eMethods.

Data. Data for this study were obtained from the Michigan Sickle Cell Data Collection (MiSCDC) program and publicly available Early and Periodic Screening, Diagnostic and Treatment (EPSDT) Annual Reporting Data Files from the Centers for Medicare & Medicaid Services (CMS) (Form CMS-416). MiSCDC gathers health information from multiple data sources about people living with SCD to inform policy changes, health care services improvements, and new treatments. Use of these data was determined to be not regulated as it is a public health surveillance activity and approved by IRBs at the University of Michigan (approval number HUM00179707) and Michigan Department of Health and Human Services (approval number 202004-13-NR).

Sample. We examined children aged 1-20 years in the MiSCDC program who were continuously enrolled for 3 or more months in Michigan Medicaid in 2022. Using 2022 CMS EPSDT Annual Reporting Data Files, we also examined children ages 1-20 enrolled in Medicaid in Michigan for 3 or more months.

Variables. Dental services were identified using the following Healthcare Common Procedure Coding System (HCPCS) codes: any dental services (D0100–D9999), preventive dental services (D1000-D1999) and dental treatment services (D2000–D9999).

© 2025 Kranz A et al. *JAMA Network Open*.
