# Supplementary material for: Sickle Cell Disease and Dental Care Access Among Medicaid-Enrolled Youths
Source: JAMA Netw Open. 2025 Sep 3;8(9):e2529849. doi: 10.1001/jamanetworkopen.2025.29849 (PMC12409594; doi:10.1001/jamanetworkopen.2025.29849)
Supplement: Supplement 2. — Data Sharing Statement [file jamanetwopen-e2529849-s002.pdf]

Data Sharing Statement

Kranz. Sickle Cell Disease and Dental Care Access Among Medicaid-Enrolled Youths. *JAMA Netw Open*. Published September 03, 2025. doi:10.1001/jamanetworkopen.2025.29849

Data
Data available: Yes
Data types: Deidentified participant data
How to access data: CMS-416 Data is publicly available: https://www.medicaid.gov/medicaid/benefits/early-and-periodic-screening-diagnostic-and-treatment
When available: beginning date: 01-16-2025

Supporting Documents
Document types: None

Additional Information
Who can access the data: CMS-416 Data is publicly available: https://www.medicaid.gov/medicaid/benefits/early-and-periodic-screening-diagnostic-and-treatment. The MiSCDC program are not publicly available because data use agreements prohibit the release of this data.
Types of analyses: CMS-416 Data is publicly available: https://www.medicaid.gov/medicaid/benefits/early-and-periodic-screening-diagnostic-and-treatment. The MiSCDC program are not publicly available because data use agreements prohibit the release of this data.
Mechanisms of data availability: CMS-416 Data is publicly available: https://www.medicaid.gov/medicaid/benefits/early-and-periodic-screening-diagnostic-and-treatment. The MiSCDC program are not publicly available because data use agreements prohibit the release of this data.
